# Gender Differences in Coronary Artery Disease: Correlational Study on Dietary Pattern and Known Cardiovascular Risk Factors

**Published:** 2013-12-01

**Authors:** Mahdi Najafi, Mehrdad Sheikhvatan

**Affiliations:** 1 Tehran Heart Center, Tehran University of Medical Sciences, Tehran, IR Iran

**Keywords:** Coronary Artery Disease, Risk Factors, Gender, Nutrition, Mediterranean Diet

## Abstract

**Background:**

The relationship between diet and cardiovascular risk factors in men and women with Coronary Artery Disease (CAD) has been the subject of recent studies. We studied a group of Iranian CAD patients to analyze any relationship between diet and CAD risk factors based on gender.

**Methods:**

In this study, 461 consecutive patients were assessed before their planned isolated coronary artery bypass graft surgery. They were interviewed to obtain the quantity and components of nutrients and micronutrients based on a validated food frequency questionnaire. Diet scores were calculated in each dietary group and the total score was reported as the Mediterranean Diet Quality Index (Med-DQI). Physical activity was assessed using International Physical Activity Questionnaire (IPAQ). Functional class, EuroSCORE and the frequency of the known risk factors in the men and women were recorded as well.

**Results:**

The women were more likely than the men to present with obesity, diabetes mellitus, hypercholesterolemia, and hypertension (all Ps < 0.001). Also, the women had higher functional class and mean of EuroSCORE (P < 0.001 and P = 0.03). Only six women (5.7%) reported to have regular physical activity. In addition, Women’s energy intake was more likely to be supplied through fat. Cereals, fruit, and vegetable consumption in both genders was within the safe recommended range, while olive and fish consumption was low in both sexes. MedDQI score was different between men and women with hypertension (P = 0.018) and obesity (P = 0.048).

**Conclusions:**

Modifiable classical risk factors for CAD, except for smoking, were more prevalent in women and were associated with their diet. Therefore, women probably need to maintain low calorie intake while improving physical activity and dietary patterns to decrease the frequency and severity of modifiable cardiac risk factors.

## 1. Background

The major cardiac risk factors, proposed in 1961 by the Framingham Heart Study, include smoking, hypertension, diabetes, hyperlipidemia, low physical activity, older age, and male gender. These risk factors have been the most important predictors of cardiovascular outcome in healthy individuals and the patients with Coronary Artery Disease (CAD) since their recognition (1). As much as CAD is still more common in men, it is no longer deemed the “man’s disease” courtesy of the report series on the importance and unique features of CAD in women in the late 90s (2). What is more, the number of women diagnosed with CAD is currently rising the world over (3). However, women are different from men regarding the pattern of coronary artery involvement, cardiac risk factors, and life style.

Lifestyle modification is the most cost-effective way to reduce the risk of CAD (4). Lifestyle changes are mainly possible through changes in diet and exercise as well as cigarette smoking. The difference in dietary patterns, eating habits, and nutrition beliefs between the two genders has been the subject of investigations for years. Studies in this field have revealed that women are more likely than men to diet or try other weight-loss practices (5). For all these investigations, however, the impact of a healthy dietary regimen and physical activity on men’s and women’s cardiovascular risk factors and outcome has yet to be clarified. Furthermore, it is not fully known whether dietary habits are as effective in treatment of the patients with severe CAD as in the prevention of CAD (6).

With regard to the geographical trends in sex differences (7), evaluation of dietary patterns and CAD risk factors in women and men in every region can enhance our knowledge about the prevention of this disease. The Mediterranean diet gained widespread recognition in 1990s as a type of diet with the strongest evidence for beneficial effects on cardiovascular health (8). Be that as it may, there are still debates on the mechanisms of action and the influence that this kind of diet may exert on the CAD risk factors (9,10). The present study aimed to analyze any relationship between the differences in CAD risk factors in both sexes and diet and calorie intake in a group of severe CAD patients at Tehran Heart Center, Tehran, Iran.

## 2. Patients and Methods

The patients with CAD who were candidates for isolated coronary artery bypass graft surgery at Tehran Heart Center were recruited consequently to be evaluated in this cross sectional study during a five-month period. The study sample size was calculated using the formula for comparing two proportions: n = [(Z_α/2 _+ Z_β_)^2^ × {(p1 (1-p1) + (p2 (1-p2))}]/(p1 - p2)^2^ where p1 is the proportion of the women with low quality Mediterranean regimen (0.3), p2 is the proportion of the men with low quality Mediterranean regimen (0.25), α error = 0.05, and power = 80% (1-β). Accordingly, a 125-subject sample size was determined for the study (125 in each group). We also assumed 20% loss (125 + 25) and as men with CAD are twice as women (150 + 300), the final sample size of 450 was considered for the study.

The study was approved by the Ethics Committee of Tehran University of Medical Sciences, and all the participants signed informed consent forms in accordance with the requirements of this university. Nutritional status was assessed using the Food Frequency Questionnaire (FFQ), which has been previously validated in Iran (11), and a 24-hour dietary recall questionnaire to record the types, amounts, and frequencies of the consumed foods. The FFQ consisted of a list of foods with standard serving sizes commonly consumed by Iranians (12). The patients were asked to report the frequency of their consumption of a given serving of each food item during the previous year on a daily (e.g. bread), weekly (e.g. rice and meat), or monthly (e.g. fish) basis. The reported frequency for each food item was then converted to a daily intake.

Additionally, the diet score was calculated on the basis of the Mediterranean Diet Quality Index (Med-DQI) (Table 1). This type of index suits the dietary habits of the Iranian population with lower alcohol and high fruits and vegetables (13). The index assigns a score of 0, 1, or 2 according to the daily intake of each of the seven components, and the final score is reported as the sum of all the nutrient scores ranging from 0 to 14. A lower score on this index indicates a better nutrition quality; the scores from 0 to 4 connote the highest quality, while the scores between 11 and 14 signify the lowest quality. It should be mentioned that the patients did not receive any educational content in any form about their nutrition.

**Table 1.** Mediterranean Dietary Quality Index (Med-DQI) Scores

Scoring	0	1	2
**Saturated fatty acids, % energy**	< 10	10 – 13	> 13
**Cholesterol, mg**	< 300	300 – 400	> 400
**Meats, g**	< 25	25 – 125	> 125
**Olive oil, mL**	> 15	15 – 5	< 5
**Fish, g**	> 60	60 – 30	< 30
**Cereals, g**	> 300	300 – 100	< 100
**Vegetables + fruits, g**	> 700	700 – 400	< 400

The presence of any cardiac risk factor was determined through interview, physical examination, and laboratory data, whichever was applicable. Physical activity was assessed using International Physical Activity Questionnaire (IPAQ). Moreover, the New York Heart Association (NYHA) functional class and EuroSCORE were determined based on the standard definitions.

The results are reported as mean ± Standard Deviation (SD) for the quantitative variables and percentages for the categorical ones. The study groups were compared using Student t-test for the continuous variables and the chi-square test or the Fisher exact test, if required, for the categorical variables. Besides, a P value ≤ 0.05 was considered as statistically significant. All the statistical analyses were performed using the SPSS statistical software, version 13.0 (SPSS Inc., Chicago, IL., USA) for Windows.

## 3. Results

The present study was conducted on 461 patients (range = 35 to 80 years old; 77% men and 23% women). The demographic characteristics and clinical data of the participants are summarized in Table 2. According to the results, the women had lower educational levels compared to the men. However, the frequency of cigarette smoking, opium addiction, and recent myocardial infarction were higher in the men, while the women were more likely to present with the history of obesity, diabetes mellitus, hypercholesterolemia, and hypertension. Also, the women had higher functional class and mean of EuroSCORE.

**Table 2.** Demographic Characteristics and Clinical Data of the Study Patients[Table-fn fn6746]

Characteristics	Men (n = 357)	Women (n = 104)	*P* value
Age (year)	58.11 ± 9.38	59.12 ± 7.13	0.313
Body mass index (kg / m2)	26.64 ± 3.41	29.11 ± 4.69	< 0.001
**Education level:**			
Primary	50 (14.0)	50 (48.1)	< 0.001
Secondary	155 (43.4)	39 (37.5)	
Higher	152 (42.6)	15 (14.4)	
Family history of CAD	165 (46.2)	49 (47.1)	0.872
Current cigarette smoking	166 (46.5)	(2.9)	< 0.0013
Hypercholesterolemia	233 (65.3)	89 (85.6)	< 0.001
Diabetes Mellitus	117 (32.8)	65 (62.5)	< 0.001
Hypertension	140 (39.2)	78 (75.0)	< 0.001
Cerebrovascular disease	14 (3.9)	3 (2.9)	0.621
Peripheral vascular disease	76 (21.3)	40 (38.5)	< 0.001
Recent myocardial infarction	198 (55.6)	35 (33.7)	< 0.001
Ejection fraction (%)	48.08 ± 9.89	52.25 ± 9.48	< 0.001
**Functional class:**			
I	146 (40.9)	18 (17.3)	< 0.001
II	175 (49.0)	57 (54.8)	
III	36 (10.1)	29 (27.9)	
EuroSCORE	2.02 ± 2.14	2.66 ± 1.81	0.003
Physical activity	105 (29.8)	10 (10.7)	< 0.001
Regular Physical activity	84 (23.9)	6 (5.7)	< 0.001
**Coronary vessels involvement:**			
Single-vessel disease	12 (3.4)	8 (7.7)	0.152
Two-vessel disease	78 (21.8)	20 (19.2)	
Three-vessel disease	267 (74.8)	76 (73.1)	
**Laboratory indices:**			
Last fasting blood sugar (mg / dL)	104.16 ± 32.31	115.35 ± 35.86	0.005
Last creatinine (mg / dL)	1.29 ± 0.24	1.09 ± 0.20	< 0.001
Triglyceride (mg / dL)	173.26 ± 95.30	173.86 ± 66.94	0.943
Cholesterol (mg / dL)	156.64 ± 42.91	172.46 ± 44.16	0.001
High density lipoprotein (mg / dL)	39.24 ± 8.21	43.63 ± 8.44	< 0.001
Low density lipoprotein (mg / dL)	85.09 ± 38.91	94.33 ± 41.59	0.045
Lipoprotein (a) (mg / dL)	31.31 ± 25.74	32.38 ± 29.49	0.739
Albumin (g / dL)	4.67 ± 0.33	4.62 ± 0.31	0.187

Abbreviations: CAD, Coronary Artery Disease; Data are presented as mean ± SD or number (percentages)

The findings of this study showed that physical activity was low among the women (15 women, 10.7%) and only six women had regular physical activity (Table 2). Therefore, the role of physical activity was not taken into account in comparison of men and women.

Furthermore, no significant difference was found between the two genders with respect to the number of defected coronary arteries. Among the laboratory parameters, the means of fasting blood glucose, cholesterol, and low-density lipoprotein were higher in the women compared to the men. However, the serum concentrations of triglyceride and lipoprotein (a) were similar between the two genders.

The total energy intake was higher in the men compared to the women (Table 3). Women’s energy intake was more likely to be supplied through fat (Table 4). In addition, a significant difference was observed between the men and women regarding three out of the seven components of Mediterranean dietary pattern scores; i.e., saturated fatty acid, cholesterol, and cereals (Table 4). Nevertheless, consumption of cereals, fruits, and vegetables were within the safe recommended range in both genders. On the other hand, olive oil and fish consumption was low in both sexes. No association was found between MedDQI score and the risk factors in the two sexes (Table 5). 

**Table 3. tbl10341:** Daily Nutrient Intake in the two Genders[Table tbl10341]

Nutrients Intakes	Men (n = 357)	Women (n = 104)	*P* value
**Energy (Kcal / d)**	3013.02 ± 1320.34	2333.36 ± 896.07	< 0.001
**Carbohydrate (gr / d)**	463.77 ± 230.89	350.11 ± 150.86	< 0.001
**Protein (gr / d)**	108.60 ± 47.46	87.34 ± 28.91	< 0.001
**Fiber (gr / d)**	40.07 ± 21.85	33.50 ± 13.53	< 0.001
**Total fat (gr / d)**	87.77 ± 43.66	71.89 ± 32.91	< 0.001
**Saturated fat (gr / d)**	33.37 ± 16.68	28.09 ± 14.69	0.002
**Monounsaturated fat (gr / d)**	33.67 ± 17.46	26.85 ± 12.75	< 0.001
**Polyunsaturated fat (gr / d)**	20.93 ± 11.89	15.69 ± 6.75	< 0.001

Data are presented as mean ± SD

**Table 4.** Mediterranean Dietary Scores in the two Genders[Table-fn fn6748]

Dietary Group	Men (n = 357)	Women (n = 104)	*P* value
**Saturated fatty acid**			0.005
0 (n = 225)	53.4	36.1	
1 (n = 168)	33.0	46.2	
2 (n = 68)	13.6	17.6	
**Cholesterol**			
0 (n = 332)	67.9	83.2	0.004
1 (n = 69)	16.4	10.9	
2 (n = 60)	15.7	5.9	
**Meats**			
0 (n = 156)	31.2	41.2	0.077
1 (n = 277)	61.7	55.5	
2 (n = 28)	7.1	3.4	
**Olive**			
0 (n = 50)	13.0	5.0	0.059
1 (n = 146)	30.9	33.6	
2 (n = 265)	56.2	61.3	
**Fish**			
0 (n = 59)	13.9	10.1	0.540
1 (n = 95)	20.7	20.2	
2 (n = 307)	65.4	69.7	
**Cereal**			
0 (n = 319)	75.9	50.4	< 0.001
1 (n = 133)	22.8	45.4	
2 (n = 9)	1.2	4.2	
**Fruits and vegetables**			
0 (n = 372)	81.2	79.0	0.726
1 (n = 75)	16.0	16.8	
2 (n = 14)	2.8	2.8	

Data are presented as percentages

**Table 5.** Mediterranean Diet Quality Index (Med-DQI) Score in Men and Women in Different Groups of Coronary Artery Disease Risk Factors[Table-fn fn6749]

Risk Factor Group	Men (n = 357)		Women (n = 104)	
	Final Score	*P* value	Final Score	*P* value
**Total**	5.26 ± 2.07		5.61 ± 1.77	0.095
**Age > 60**	5.20 ± 2.06	0.700	5.71 ± 1.93	0.576
**Age ≤ 60**	5.26 ± 2.07		5.52 ± 1.61	
**Diabetic**	5.26 ± 2.29	0.986	5.65 ± 1.76	0.782
**Non-diabetic**	5.25 ± 1.94		5.55 ± 1.80	
**Smoker**	5.31 ± 2.06	0.654	4.67 ± 1.16	0.349
**Non-smoker**	5.21 ± 2.07		5.64 ± 1.78	
**Hypertensive**	5.04 ± 2.22	0.121	5.73 ± 1.77	0.283
**Normotensive**	5.40 ± 1.94		5.34 ± 1.75	
**Hyperlipidemic**	5.17 ± 2.03	0.309	5.57 ± 1.81	0.480
**Normolipidemic**	5.42 ± 2.13		5.93 ± 1.44	
**Obese**	5.11 ± 1.98	0.526	5.83 ± 1.84	0.212
**Non-obese**	5.29 ± 2.09		5.42 ± 1.70	

Data are presented as mean ± SD

## 4. Discussion

The present study showed differences between the two genders regarding the daily intake of dietary components. Besides, most of the general risk factors of CAD were more prevalent among the women in comparison to the men. Moreover, two values of the outcome measure; i.e., EuroSCORE and functional class, were higher in the women. The main question is to what extent diet can influence the CAD risk factors and outcome.

The results of the present study revealed Med-DQI to be low in both men and women (Table 5). This reflects a pattern of a healthy diet for the heart in both sexes in our study. As expected, the energy intake of the women was lower than that of the men. We know from the Harris Benedict equation (A Biometric Study of Human Basal Metabolism. Harris JA and Benedict FG.Proceedings of the National Academy of Sciences. 1918; 4 (12) : 370 - 373) that the energy requirement in all ages and activity groups is higher in men compared to women. The last statistical update of American Heart Association (AHA) about heart disease and stroke emphasizes that energy intake in women -has increased significantly but still- is lower compared to men in the recent years (14). We have previously shown that the women with CAD have higher energy intake than expected and tend to underreport their energy intake (15). The women’s energy imbalance in our study is supported by the high rates of obesity, hyperlipidemia, and glucose intolerance as well as low level of physical activity among the women compared to the men.

More comorbid conditions in the women than in the men at the time of presentation with cardiovascular disease in our study have been also documented before (16, 17). In the last decade, attempts have been made to distinguish lifestyle and specific dietary factors that may increase the cardiovascular risk. Indeed, the frequency and characteristics of the risk factors among the women with coronary disease look like different from those of men. In fact, women are more likely to have modifiable risk factors that are related to life habits, such as obesity and physical inactivity (2, 18, 19).

Low educational level parallel to unusual features of CAD in women may worsen the current situation of knowledge about CAD in women. The lower level of education among the women compared to the men in this study suggests that they are more probable to ignore the modifiable cardiac risk factors and refuse to comply with healthy diet and physical activity guidelines. A report from the AHA demonstrated that awareness about the CAD risk factors as well as the knowledge about the need for emergency care in cardiovascular events is still low around the globe (2). These risk factors are modifiable by a planned educational health program. National Cholesterol Education Program suggests in its guideline, the Adult Treatment Panel III (ATP III), that therapeutic lifestyle changes are an essential modality even in high-risk patients who have lifestyle-related risk factors, such as obesity and hyperlipidemia (20). Women’s education about heart-healthy diets should be focused on simple healthy eating and exercise habits. Stampfer et al. showed that among the middle-aged women without known vascular disease, adherence to healthy diet, regular physical activity, and avoidance of smoking and obesity were associated with a risk of CAD of less than half that of age-matched women without these lifestyle characteristics (21). Importantly, favorable effects of lifestyle were still evident after adjustment for known CAD risk factors, such as age, family history, hypertension, hypercholesterolemia, and menopausal status.

The present study also demonstrated that the majority of the patients followed the Mediterranean dietary pattern, including low cholesterol and high cereal, fruit, and vegetable contents, while the rate of olive and fish consumption was low (Table 4). Except for this unsatisfactorily low consumption of fish and olive, the patients had moderately high adherence to the Mediterranean dietary pattern according to the Med-DQI score (22). As dietary patterns in various populations depend on geography (7), people who live in the Mediterranean region are fortunate to have a heart-healthy dietary pattern. Our findings chime in with this observation insofar as the Med-DQI reflected a nearly high diet quality in both genders. Differences between men and women concerning the dietary pattern in some studies underscore the possible role of nutritional habits in the gender differences encountered in CAD (22-24). Reports from the Iranian healthy population attribute higher prevalence of the risk factors in women to nutritional patterns (25).

Recent studies have shown that adherence to a Mediterranean regimen is promising in prevention from CAD risk factors (26). In this investigation, no significant difference was observed between the groups of patients with and without any of the major risk factors regarding the Med-DQI score (Table 5), suggesting that adherence to a healthy diet by itself may not change the outcome, at least in the patients with severe CAD who are CABG candidates. This finding was confirmed in a similar study on adherence to the DASH eating plan in a group of patients with severe CAD (27). That study demonstrated moderate adherence to the DASH diet in the CAD patients who were candidates for coronary artery bypass. In addition, no difference was observed between the hypertensive and normotensive patients as well as between the men and women with regards to the scores.

Low level of physical activity overall and in women in this study may be another reason for our failure to finding differences in Med-DQI score between the groups of patients with and without any of the major risk factor (Table 2). It is evident from the literature that both diet and physical activity are involved in the etiology of cardiac risk factors (28, 29). Besides, the current knowledge shows that an imbalance between energy intake and expenditure has adverse effects on the patients’ risk factors and quality of diet (29, 30).

Our study showed that although the women’s adherence to the heart-healthy Mediterranean diet was acceptable, they were more likely to suffer from energy imbalance probably due to low level of physical activity. Therefore, nutrition education programs in the women with CAD in the Mediterranean region should be mainly focused on creating a balance between energy demand and intake.

Regarding the modifiable nature of the women’s risk factors in this study, such as obesity, hyperlipidemia, and diabetes, it is possible to improve the patients’ long-term outcome by having a healthy diet with lower calorie intake and consumption of higher values of marine food and liquid vegetable oil. Yet, preventive measures are likely to be more effective when implemented as soon as possible before or in the early stages of CAD.

### Study Limitations

We didnot analyze the relationship between physical activity and diet and cardiac risk factors in men and women as the level of regular physical activity was very low among the Iranian patients overall and particularly in women. Thus, further studies are suggested to evaluate the effects of physical activity in a larger number of participants.

Moreover, we did not know the dietary regimen of our patients before being candidates of CABG and/or becoming diabetic. Therefore, we can not precisely claim that their current dietary regimen is different from the regimen they followed before becoming patient or not. We also do not know whether or not there is any difference between the patients regarding their knowledge about healthy diet or healthy life style.
